# Ion Beam Assisted Deposition of Thin Epitaxial GaN Films

**DOI:** 10.3390/ma10070690

**Published:** 2017-06-23

**Authors:** Bernd Rauschenbach, Andriy Lotnyk, Lena Neumann, David Poppitz, Jürgen W. Gerlach

**Affiliations:** 1 Leibniz Institute of Surface Modification, Permoserstr. 15, 04318 Leipzig, Germany; andriy.lotnyk@iom-leipzig.de (A.L.); elena.nejman@mail.ru (L.N.); david.poppitz@imws.fraunhofer.de (D.P.); juergen.gerlach@iom-leipzig.de (J.W.G.); 2 Felix-Bloch Institute for Solid State Physics, Universität Leipzig, Linnéstraße 5, 04103 Leipzig, Germany

**Keywords:** ion beam assisted deposition, gallium nitride thin films, hyperthermal ions

## Abstract

The assistance of thin film deposition with low-energy ion bombardment influences their final properties significantly. Especially, the application of so-called hyperthermal ions (energy <100 eV) is capable to modify the characteristics of the growing film without generating a large number of irradiation induced defects. The nitrogen ion beam assisted molecular beam epitaxy (ion energy <25 eV) is used to deposit GaN thin films on (0001)-oriented 6H-SiC substrates at 700 °C. The films are studied in situ by reflection high energy electron diffraction, ex situ by X-ray diffraction, scanning tunnelling microscopy, and high-resolution transmission electron microscopy. It is demonstrated that the film growth mode can be controlled by varying the ion to atom ratio, where 2D films are characterized by a smooth topography, a high crystalline quality, low biaxial stress, and low defect density. Typical structural defects in the GaN thin films were identified as basal plane stacking faults, low-angle grain boundaries forming between w-GaN and z-GaN and twin boundaries. The misfit strain between the GaN thin films and substrates is relieved by the generation of edge dislocations in the first and second monolayers of GaN thin films and of misfit interfacial dislocations. It can be demonstrated that the low-energy nitrogen ion assisted molecular beam epitaxy is a technique to produce thin GaN films of high crystalline quality.

## 1. Introduction

GaN thin films are promising candidates for many optoelectronic, high-temperature, and high-power applications [1]. GaN is almost commonly observed as the stable wurtzite 2H polytype (w-GaN), but can also crystallize in a metastable zinc-blende 3C structure (z-GaN) or the metastable (fcc) rock salt structure (c-GaN). Several physical and chemical technologies (e.g., molecular beam epitaxy or metal-organic chemical vapor deposition) are applied to synthesize GaN films. A general unsolved problem of the manufacturing of such films, which restricted the application of GaN films in optoelectronic and electronic devices, is the crystalline quality. Especially, the high concentration of background n-type carrier induced by lattice defects and the lack of the highly lattice-matched substrates commonly requires thick films up to 10 µm, because the defect concentration is reduced with increasing film thickness. Consequently, deposition technologies are wanted, which are capable to deposit thin GaN films with high crystalline quality.

Technologies which are based on the low-energy ion-solid surface interaction are of particular significance in many fields of thin film technology, including sputter deposition, smoothing, cleaning and etching, ion beam lithography, depth profiling and formation of nanostructures. The ability of low-energy ions (less than some keV) to influence the nucleation of thin films, crystallinity, morphology, and defect concentration, as well as the surface topography, can provide significant modification of the thin film properties [1]. 

In conjunction with conventional deposition techniques, low-energy ions can assist the modifying film structure and composition. The ion beam assisted deposition (IBAD) refers to all those using energetic ions to change the thin film properties and/or modify its structure during the deposition. Ion energy and flux, as well as the arrival rate of ions to deposited atoms (ion to atom ratio, I/A) are the crucial parameters to control the film structure and properties. In almost all experiments and ion beam technologies to fabricate thin films, ions with energy between 1 keV and 10 keV are utilized, although it is well-known that an increase of the ion energy leads to a significant increase of the defect concentration in the growing film. Consequently, a modern trend is to use so-called hyperthermal ions with kinetic energy smaller than 100 eV to modify the properties of thin films [2,3].

Here, a special variant of the IBAD technology, the ion beam assisted molecular beam epitaxy (IBA-MBE) is deployed to prepare thin GaN films with high crystalline quality. It could be demonstrated, that this method is very successful for direct growth (i.e., without buffer layer) of epitaxial GaN films [4]. In IBA-MBE, low energetic nitrogen ions (<100 eV) are delivered to the surface of the growing thin film during the deposition process. Subsequently, the surface mobility of the landing adatoms is enhanced and synthesis of GaN thin films with a low density of extended defects is possible. Since the overall quality of GaN thin films is strongly influenced at the initial stage of thin film formation, a detailed knowledge on the real structure of the GaN film/6H-SiC substrate interface and of the first few atomic layers is essential for optimization of deposition conditions and for identifying an application potential of IBA-MBE for GaN growth.

In this article, results are presented on the crystalline structure of two-dimensional (2D) and three-dimensional (3D) GaN films on 6H-SiC(0001) prepared by IBA-MBE with hyperthermal nitrogen ions, determined using reflection high-energy electron diffraction, X-ray diffraction, scanning tunnelling microscopy, and high-resolution transmission electron microscopy. The optimum conditions for epitaxial growth and high crystalline quality are discussed in terms of the nitrogen ion to gallium atom ratio and a mechanism of misfit accommodation at the GaN/6H-SiC interface are reported.

## 2. Results and Discussion

### 2.1. Growth Characteristics

**Figure 1:**
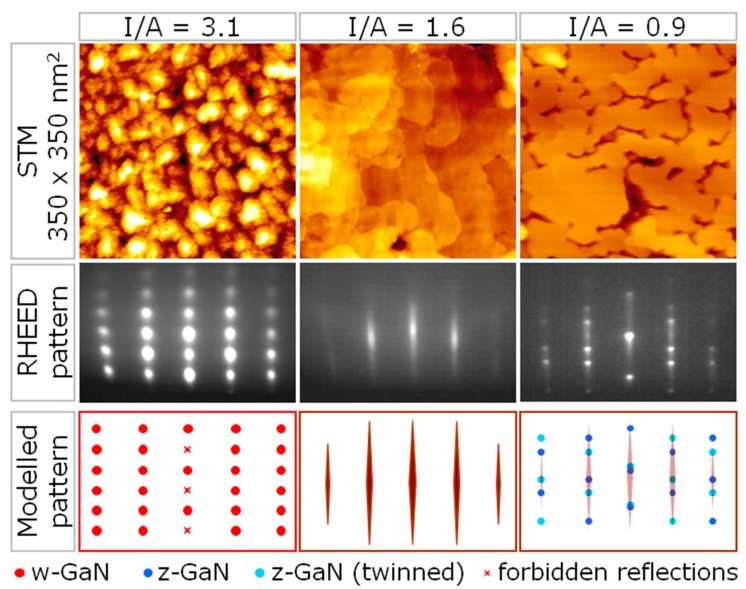
STM images, corresponding RHEED patterns and modelled patterns for ca. 10 nm thick GaN films deposited by IBA-MBE with different I/A ratios. STM: scanning tunneling microscopy; RHEED: reflection high-energy electron diffraction; IBA-MBE: ion beam assisted molecular beam epitaxy; I/A: ion to atom ratio.

Keeping the previously optimized parameters nitrogen ion energy, nitrogen ion flux, and 6H-SiC(0001) substrate temperature constant (see Section 4), the nitrogen ion to gallium atom flux ratio I/A was varied for the epitaxial GaN thin film deposition using IBA-MBE by solely altering the gallium atom flux. In a combined in situ reflection high-energy electron diffraction (RHEED)/in vacuo scanning tunneling microscopy (STM) study it was already reported by the authors [4] that the growth characteristics and the resulting film properties are dependent on the I/A ratio, i.e., on the average number of impinging hyperthermal nitrogen ions per deposited Ga atom. In particular, it was found that the growth mode of the films relies crucially on the chosen I/A ratio. While three-dimensional 3D growth was dominant for I/A ratios higher than 1.9, the preferred 2D growth was accomplished only for I/A ratios below. This is of major importance, as other film properties are defined, or at least influenced, by the film growth mode. The growth rate for 3D growth at I/A = 3.1 was 0.7 nm/min and the for 2D growth at I/A = 1.6 was 1.3 nm/min, repectively. In the present study, key aspects of this influence shall be addressed in more detail. Exemplarily, in Figure 1 the surface topography of ca. 10 nm thick GaN films obtained from STM, as well as the corresponding final RHEED patterns, are depicted for different I/A ratios. In the case of I/A = 3.1, the topography shows islands, the corresponding RHEED pattern consists of spot-like reflections, which indicates 3D growth of (0001)-oriented w-GaN. For GaN films deposited with I/A = 1.6, the topography is comparably flat with wide terraces and the RHEED pattern now consists of narrow, streak-like reflections, which are typical for 2D growth. For lower I/A ratios, this 2D growth is disturbed by secondary nucleation of (111)-oriented z-GaN islands, as indicated by the diffraction spots in the corresponding RHEED pattern. These islands appear in twinned form, i.e., they are rotated against each other around the [111] direction. For clarity, the arrangement of reflections in the RHEED patterns is depicted in the modelled patterns shown in the third row of Figure 1.

**Figure 2:**
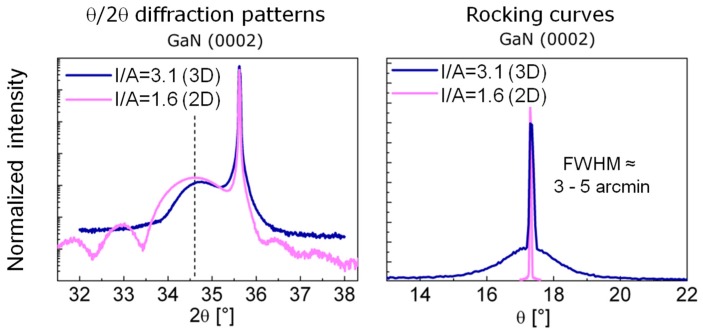
Comparison of XRD patterns and rocking curves of ca. 10 nm thick GaN films deposited by IBA-MBE with different I/A ratios exhibiting either 3D or 2D growth. The dashed vertical line marks the literature value (c = 0.5185 nm) for bulk w-GaN.

In the following, two types of ca. 10 nm thick GaN films, representative for 3D (I/A = 3.1) and 2D (I/A = 1.6) growth, will be compared regarding structural properties and film growth characterstics. The θ/2θ diffraction spectra of such samples (see Figure 2, left) show the sharp 6H-SiC(0001) reflection at 35.67° and much broader GaN(0002) reflections, due to the small film thickness, near the expected position (34.56°) indicated by the vertical dashed line. The maximum of the diffraction curve from the 3D grown film (blue) is slightly shifted to higher diffraction angles, which indicates a slight reduction of the c-axis length. The maximum of the curve for the 2D grown film (magenta) is situated at the literature value position [5]. As the surface topography of the 2D grown films is flat, i.e., the film thickness homogeneity is high over the whole film, the reflection is accompanied by film thickness fringes. As a measure for the crystalline quality of the films, rocking curves of the GaN(0002) reflections were recorded. The rocking curve of the 3D grown film in Figure 2 consists of two contributions, a narrow peak with a full-width at half height (FWHM) of 5 arcmin together with a broad peak with a FWHM of larger than 1°. Accordingly, such films consist of crystallites with a high order of orientation as well as of highly misaligned crystallites. In contrast, the rocking curve of the 2D grown film is one sharp peak with a FWHM of 3 arcmin being indicative of a high order of orientation, as can be expected from high crystalline quality epitaxial growth.

**Figure 3:**
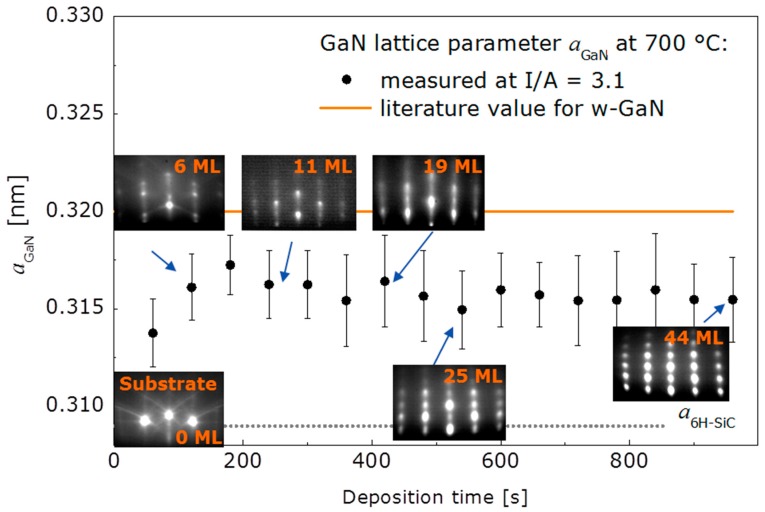
Evolution of in-plane lattice periodicity with deposition time for 3D GaN film growth. Corresponding RHEED patterns are given for representative nominal film thicknesses.

**Figure 4:**
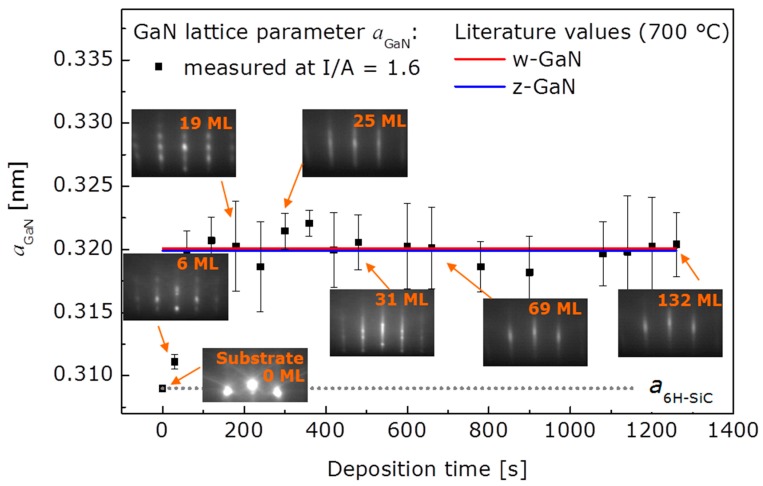
Evolution of in-plane lattice periodicity with deposition time for 2D film growth. Corresponding RHEED patterns are given for representative nominal film thicknesses.

**Figure 5:**
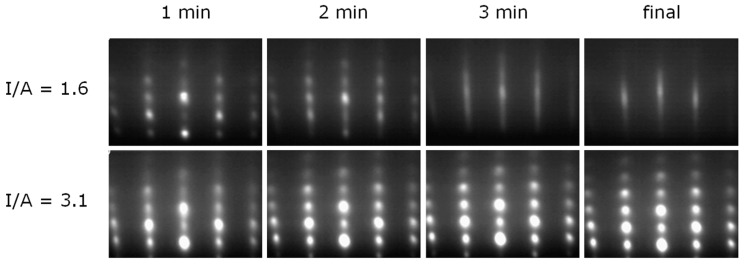
Evolution of RHEED patterns with deposition time for 2D growth (top) and 3D growth (bottom).

**Figure 6:**
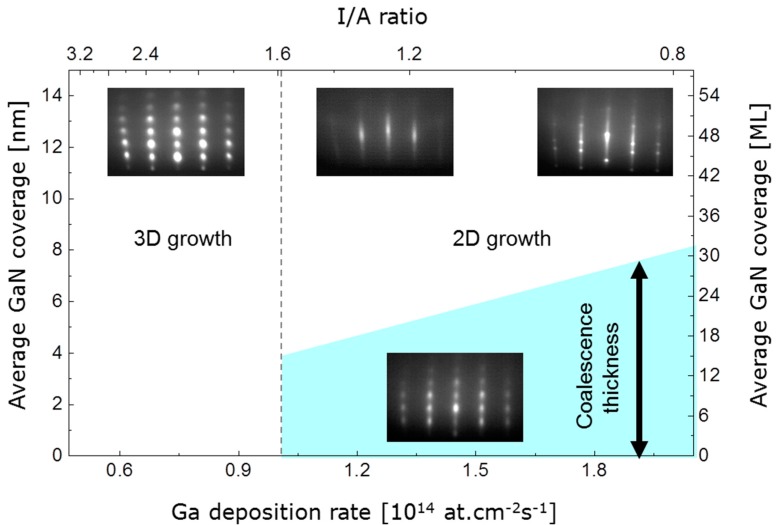
Schematic IBA-MBE GaN growth parameter map with experimentally found regions of 3D growth, as well as of 2D growth before and after film coalescence.

In order to examine the initial growth stage of 3D and 2D growth, series of RHEED patterns were recorded, allowing for the nominal film thickness dependent determination of the in-plane lattice spacing. Starting with the bare 6H-SiC(0001) substrate as a reference, the evolution of the lattice parameter a_GaN_ with deposition time is illustrated in Figure 3 for the case of 3D growth. The dotted line marks the substrate reference value. Corresponding RHEED patterns are given in addition. In the beginning of 3D growth, the in-plane periodicity of the GaN islands increases and reaches the final state already after a nominal thickness of six monolayers (ML) of GaN. The final value around 0.316 nm lies below the value expected for bulk w-GaN at the growth temperature 700 °C (drawn through line in the Figure 3). From beginning on the RHEED patterns of the growing film exhibit spot-like reflections typical for 3D growth. In comparison, the in-plane periodicity for 2D growth (see Figure 4) evolves also with an increase in the beginning, reaching the final state within the first 6 ML, too, but the final value is in good agreement with the value expected for bulk w-GaN at the growth temperature of 700 °C. This means that the 2D grown GaN films are relaxed within the error of the measurement as already derived from the X-ray diffraction (XRD) measurements. The RHEED patterns evolution for 2D growth is more complex than for 3D growth. For reasons of clarity this is illustrated in Figure 4. According to the upper series of RHEED patterns in Figure 5, growth starts with the formation of well-oriented islands, indicated by sharp, spot-like reflections. This initial island growth undergoes a rapid transition to 2D growth (I/A = 1.6), as can be concluded from the change to streak-like reflections in the patterns for longer deposition times. In the shown case, this transition is accomplished after already 3 min of deposition. For 3D growth (I/A = 3.1) however, the RHEED patterns start with broad, spot-like reflections and the patterns recorded after 2 min, 3 min, and at the end of the deposition do not differ from each other anymore. This means, that while further details about the growth evolution for 3D grown films cannot be derived from the RHEED pattern, for the case of 2D growth, the crucial film growth stage of film coalescence could be observed. This is summarized in Figure 6, which shows, schematically, the region of 3D growth, as well as the regions of 2D growth in the stage before and after film coalescence. It was found that within the region of 2D growth the coalescence thickness increased with the decreasing I/A ratio in the range from about 4 to 8 nm of GaN coverage. The achievement of such small coalescence thicknesses proves the capabilities of the IBA-MBE technique in comparison to other prominent GaN film growth techniques regarding thin and ultrathin epitaxial GaN films of high crystalline quality. In the literature, reported coalescence thicknesses for GaN film growth on 6H-SiC(0001) substrates are 25 nm [6] and 20–70 nm [7] for plasma-assisted molecular beam epitaxy, 70–140 nm for metalorganic vapor phase epitaxy using a thin AlN buffer layer [8], and more than 100 nm for hydride vapor phase epitaxy [9].

### 2.2. Microstructure and Defects

**Figure 7:**
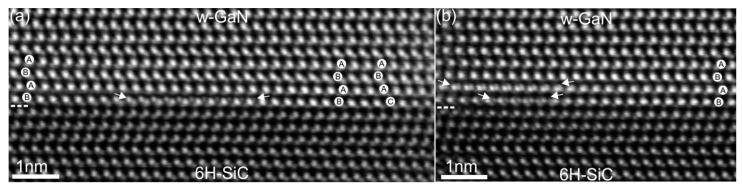
Atomic-resolution LAADF-STEM images of the GaN/SiC interfaces with double positions of Ga atomic columns in the first GaN layer in (**a**) and in two GaN layers (first and second) in (**b**). The layers are marked by arrows in (a,b). Brighter spots represent Ga and Si atomic columns whereas darker spots are N and C atomic columns in the GaN and SiC lattices, respectively. The dashed lines mark the GaN/SiC interface. The viewing direction is 6H-SiC || w-GaN. LAADF-STEM: low-angle annular dark-field scanning transmission electron microscopy.

The characterization of GaN thin film microstructure starts from the GaN/SiC interface by Cs-corrected scanning transmission electron microscopy (STEM). Among various structural methods, this is a very powerful technique and can provide information about the local structure of functional materials in real-space with resolution down to the picometer scale [10]. Atomic-resolution low-angle annular dark-field scanning transmission electron microscopy (LAADF-STEM) images of the GaN/SiC interface in $\left[2\bar{1}\bar{1}0\right]$ viewing directions of GaN and SiC lattices are shown in Figure 7. Qualitative interpretation of such STEM images is quite straightforward since the image intensities are proportional to the atomic number, as ~Z^1.8^ and the atomic columns with higher average Z number in the LAADF-STEM or high-angle annular dark-field scanning transmission electron microscopy (HAADF-STEM) images appear brighter than the columns with lower average Z number [11]. The GaN/SiC interface in Figure 7 is epitaxial and atomically flat. Due to the LAADF imaging conditions [10], the light and heavy elements are seen at the GaN/SiC interface. A large number of basal plane-like stacking faults (SFs) of intrinsic type originate directly at the GaN/SiC interface. However, regions with the hexagonal stacking sequence of the w-GaN were also found. The SFs do not propagate through the whole layer and are not bounded by partial dislocations. The SFs are mainly concentrated in the first three layers of GaN. In addition, gallium atomic columns with double positions were observed in the first and the second layers of the GaN (description shown latter in the text). The misfit or edge dislocations are not seen in this viewing direction since they are inclined. To image the core structure of these dislocations, the STEM specimen was tilted by 30° around the GaN [0001] axis. It should be noted that no threading dislocations were identified by **g·b** analysis of dislocations using **g** = 0002 and **g** = $01\bar{1}0$ viewed along the GaN $\left[2\bar{1}\bar{1}0\right]$ zone axis (**b** is the Burgers vector).

The averaged Ga to Ga distance in the first GaN layer calculated in the areas with the hexagonal stacking sequence of the w-GaN along the $\left[01\bar{1}0\right]$ GaN direction was estimated to be about 0.272 nm, which is 1.5% smaller compared to the calculated distances in the second layer and to the measured distances of defect-free GaN close to the surface (0.276 nm). The averaged distance in SiC in the $\left[01\bar{1}0\right]$ direction was measured to be 0.266 nm which is about 2.2% smaller than the measured distances in the first GaN layer. Thus, the lattice mismatch between the SiC substrate and the GaN film is compensated in the first monolayer of the GaN. The atomic structure of the first GaN layers and of the GaN/SiC interface viewed along the $\left[01\bar{1}0\right]$ GaN zone axis is presented below.

**Figure 8:**
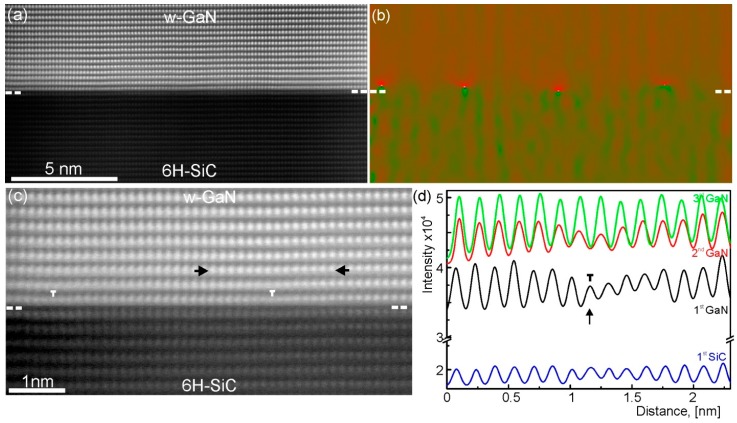
(**a**) HAADF image of GaN thin film grown on 6H-SiC substrate; (**b**) Geometrical phase analysis image calculated from (**a**). The strain fields in image (**b**) are due to dislocations; (**c**) High-resolution HAADF-STEM image of the GaN/SiC interface. Brighter spots represent Ga atomic columns in the GaN lattice, whereas darker spots are C atomic columns in the SiC lattice; (**d**) Line profiles taken along three GaN and one SiC rows. The arrows in (**c**) give the direction of line profiles shown in (**d**). The dashed lines mark the GaN/SiC interface in (**a**–**c**). The viewing direction is 6H-SiC $\left[01\bar{1}0\right]$ || w-GaN $\left[01\bar{1}0\right]$. HAADF-STEM: high-angle annular dark-field scanning transmission electron microscopy.

A high-resolution HAADF-STEM image of the GaN/SiC interface in the $\left[01\bar{1}0\right]$ viewing directions of GaN and SiC lattices is given in Figure 8a. The interface between the thin film and substrate is epitaxial. The areas containing line defects at the interface are blurred. Line profile analysis shown in Figure 8b reveals that the first Ga layers are well matched to the substrate with distances very close to the substrate distances. The edge dislocations appear in the second Ga layers. However, analysis of larger areas revealed that misfit dislocations between the thin film and SiC substrate can be also formed. Figure 8c,d shows the HAADF image and geometrical phase analyses of Figure 8c, respectively. The strain areas due to misfit and edge dislocations are seen in the image Figure 8b. Both of these types of dislocations are aligned along the $\langle 10\bar{1}0\rangle$ directions with a Burgers vector **b** = 1/3$\langle 1\bar{2}10\rangle$. These dislocations lie parallel to the interface on the (0001) basal planes [12]. The measured separation distance between these dislocations is about 4.6 nm which is close to the expected 4.77 nm calculated from bulk values. However, the arrangement of these dislocations is not uniform and the position of the dislocation cores can vary by ±1.5 nm. These results indicate that the misfit strain during thin film growth can be accommodated by two mechanisms. The first mechanism is based on the formation of misfit dislocations which are typical for heteroepitaxial growth of highly mismatched compounds. The second mechanism is differing from the known mechanisms. It is based on the formation of highly strained first monolayer adopting substrate symmetry with subsequent formation of edge dislocations in the following layers.

**Figure 9:**
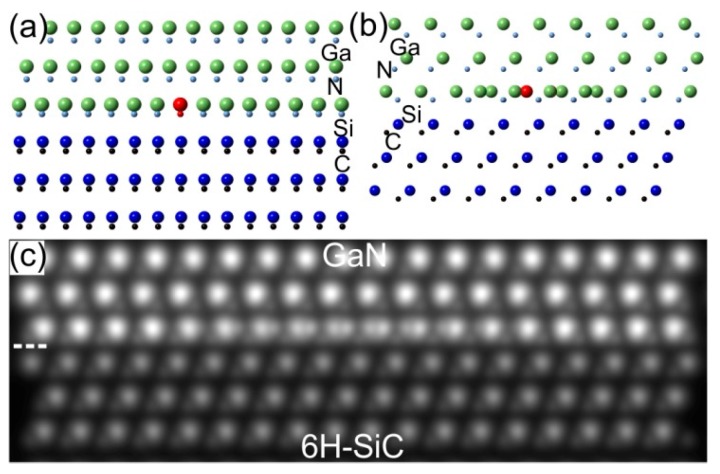
Schematic representations of the GaN/SiC interfaces (**a**) along the 6H-SiC$\left[01\bar{1}0\right]$ || w-GaN$\left[01\bar{1}0\right]$ zone axis and (**b**) along the 6H-SiC$\left[2\bar{1}\bar{1}0\right]$ || w-GaN$\left[2\bar{1}\bar{1}0\right]$ zone axis. (**c**) Simulated atomic resolution LAADF-STEM image of the GaN/SiC interface shown in (**b**) (see also Figure 7).

Schematic representations of the local structure at the GaN/SiC interface in $\left[01\bar{1}0\right]$ and $\left[2\bar{1}\bar{1}0\right]$ viewing directions are shown in Figure 9a,b. Figure 9a shows edge dislocation in the GaN whereas Figure 9b represents the defect with double Ga positions similar to the image in Figure 7. This defect can be seen as overlapping two parts of the GaN layer and can be constructed assuming Amelinckx et al.’s stacking mismatch boundary [13]. The extra plane in Figure 9a can belong either to the right or to the left part of the GaN monolayer. Image simulation based on the model of Figure 9b is shown in Figure 9c. The image contrast of the experimental image due to the defect can be reproduced well in simulated STEM images, confirming the models in Figure 9a,b. Thus, this defect is not due to steps at the 6H-SiC substrate which was discussed as the main source of the formation of Amelinckx et al.’s stacking mismatch boundary [14,15]. The formation of this defect is more likely during the growing process where the diffusing Ga and N adatoms try to match the substrate symmetry forming such overlapping regions.

**Figure 10:**
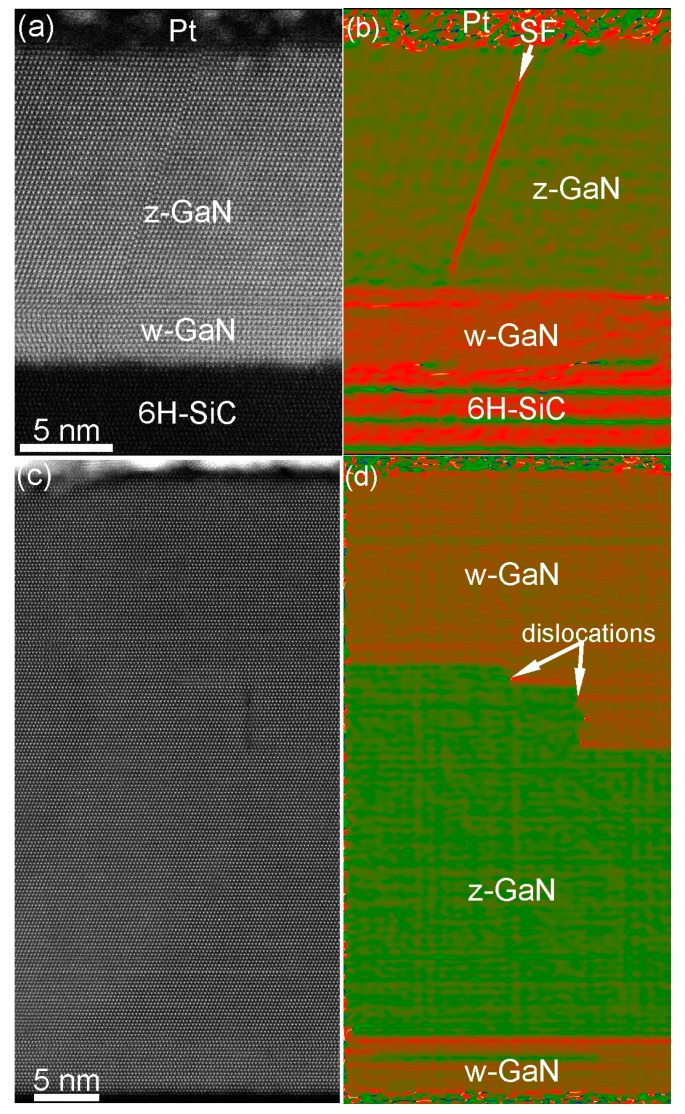
HAADF-STEM micrographs of (**a**) 18 nm and (**c**) 48 nm thick GaN thin films grown on 6H-SiC substrates. (**b**,**d**) geometrical phase processing of (**a**,**c**) for better visualization of GaN phases and defects in GaN thin films.

Above the first, second, and third GaN layers, the thin film growth proceeds by the formation of w-GaN up to a thickness of approximately 4 nm. Figure 10a–d shows HAADF-STEM images of GaN thin film with various overall thicknesses. A well-defined layer of w-GaN is presented in both samples. The typical defects in these regions were identified as basal-plane stacking faults (SFs), grain, twin, and stacking mismatch boundaries [13]. Above this 4 nm thick w-GaN layer, GaN growth continues with the formation of z-GaN. However, the thickness of this layer was varied depending on the GaN thin film thickness [13]. In Figure 10a the thickness of z-GaN is 14 nm whereas for thicker sample shown in Figure 10c the thickness of z-GaN is varying between 22 and 30 nm. Consequently, GaN thin film consisting of mainly z-GaN can be grown on top of thin w-GaN layer by IBA-MBE. Intrinsic SFs and grain boundaries are usual defects within of this z-GaN layer. Above the z-GaN layer, only w-GaN is observed after GaN thin film growth. The transition between z-GaN and w-GaN grains is smooth. The grains are connected by low-angle grain boundaries bounded by dislocations. The w-GaN layer on top of the z-GaN layer contains a lower density of planar defects comparing to the w-GaN and z-GaN layers close to the interface. Only some rarely distributed basal-plane SFs and grain boundaries were found during HAADF-HRSTEM studies.

**Figure 11:**
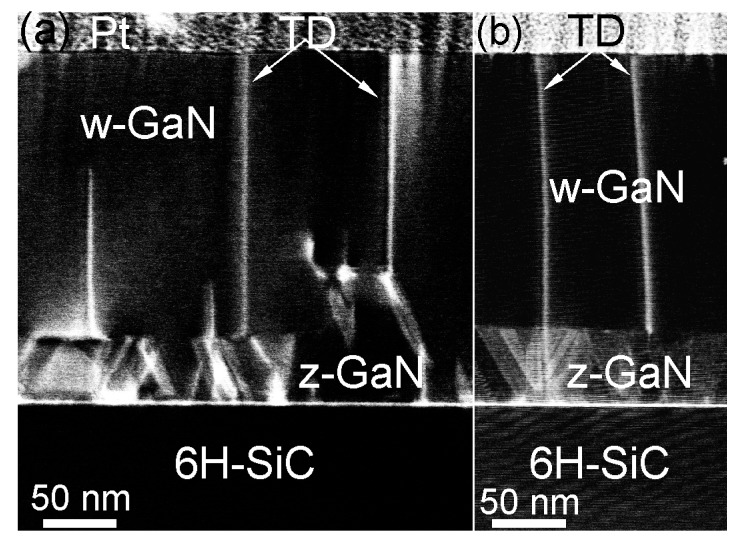
(**a**,**b**) ADF-STEM images showing threading dislocations (TDs) in GaN thin film. The bright lines at the 6H-SiC/GaN interface are due to stress caused by defects (see Figure 7).

The above described defects and GaN layer sequences were also observed in GaN thin film with a thickness of more than 200 nm. In addition, threading dislocations (TDs) are found in such thin films (Figure 11). The bright areas in Figure 11a,b are due to strain contrast caused by electron de-channeling effects at defect areas. The source of TDs was found to be different. TDs can be formed at the interface area and propagate through the whole thin film thickness. Moreover, TDs can be nucleated at pre-existing defects in z-GaN layer. The TDs can be also stopped at some thickness of GaN. Since identification of density of TDs from STEM requires large TEM specimen areas, the TDs density was first estimated from XRD. The dislocation density can be approximately estimated using D = (FWHM)^2^/4.36 × b^2^ [16], where D is the dislocation density in the film, FWHM is the FWHM value of a given XRD peak in Figure 2 in rad, and b is the length of the Burgers vector of the corresponding dislocation type. The estimated TDs dislocation density, assuming 0.05° as measured for the thickest GaN thin film (Figure 5), is 9.3 × 10^7^ cm^−2^. In addition, the overall defect density in GaN thin films was also calculated from electron channeling contrast imaging with a scanning microscope [17] and was found to be 2.9 × 10^8^ cm^−2^. Both values are comparable with defect density (10^8^–10^10^ cm^−2^) in GaN thin films grown on different substrates and by standard deposition methods [18]. 

## 3. Materials and Methods 

GaN thin films were grown by ion beam-assisted molecular beam epitaxy (IBA-MBE) consisting of three interconnected ultra-high vacuum chambers for film deposition including reflection of high-energy electron diffraction (RHEED) for in situ studies of film growth, for film analysis by Auger electron spectroscopy (AES) and low-energy electron diffraction (LEED) and for surface inspection by scanning tunneling microscopy (STM) [4]. The residual gas pressure prior deposition was 10^−7^ Pa. Ga was deposited by an effusion cell at temperatures between 950 °C and 1050 °C. In this temperature range the deposition rate Φ_Ga_ varied between 5 × 10^13^ and 2 × 10^14^ Ga atoms/cm² according to the calibration relation Φ_Ga_ = 8 × 10^7^ × exp (T_EC_/71) obtained by Rutherford backscattering spectroscopy (T_EC_ is the temperature of the effusion cell). A hollow-anode ion source, based on the principle of a constricted continuous current (DC) glow-discharge [19], was utilized to generate atomic nitrogen ions N^+^ and molecular ions N_2_^+^, where the ratio of N^+^/N_2_^+^ is roughly of order ¼. The maximum kinetic energy of these hyperthermal ions was 25 eV, and the nitrogen ion flux of 1.6 × 10^14^ ions/cm² was kept constant. 

As substrate material, super-polished (0001)-oriented 6H-SiC was chosen as the most suitable material due to its small lattice mismatch of 3.5% and quite similar thermal and mechanical properties. The temperature of the substrate during deposition was kept constant at 700 °C. This temperature was found to be the optimal temperature for growing of dense GaN thin films with high crystalline quality [4].

The deposition process was monitored in situ by reflection high energy electron diffraction (RHEED) with an electron acceleration voltage of 30 kV. The electron beam was in the $\left[2\bar{1}\bar{1}0\right]$ azimuth of hexagonal GaN and of the SiC substrate. The incidence angle of the electron beam with respect to the sample surface was ca. 2°.

The evaluation of the crystalline quality of the GaN thin films was performed by ex situ X-ray diffraction (XRD) for evaluating the average tilt and twist rotation angles of the crystallites by means of the rocking curve method. For the rocking curve (ω-scan) measurements a high-resolution diffractometer using a collimated and monochromatic Cu-Kα_1_ beam with a wavelength of 0.15406 nm was applied. The diffractometer with a four-axis goniometer allows tilt (polar angle χ, 0–88°) and rotation (azimuthal angle ϕ, 0–360°) of the sample with respect to the X-ray beam to align the sample accurately, as well as to perform texture goniometry measurements, where angular step widths of 1° in both χ and ϕ were adjusted. The rocking curve measurement (ϕ-scan) for the determination of the crystallite twist component was done with a high-resolution diffractometer equipped with an in-plane diffraction arm for in-plane measurements using a parallel beam. The in-plane ϕ-scans were performed at grazing incident beam of 0.25° to the sample surface (horizontal). 

The surface topography was studied by in situ scanning tunnelling microscopy (STM) by movement of the samples from the preparation chamber into the STEM chamber without interruption of high vacuum.

The microstructure of deposited GaN films was studied by aberration-corrected scanning transmission electron microscopy (Cs-corrected STEM). For STEM investigations, cross-sectional TEM specimens were prepared by the focused ion beam (FIB) technique. The FIB lamellae were cut out using a Ga ion beam with beam energy of 30 keV and beam current of 4 nA. The lamellae were polished to electron transparency with a 5 keV Ga ion beam at a beam current of 50 pA. To reduce the TEM specimen thickness further and to improve the surface quality, the lamellae were processed with a focused argon ion beam in a NanoMill system. Ion energies from 900 eV down to 400 eV were used to remove implanted Ga ions and amorphous regions caused by the FIB process [20]. 

STEM work was performed with a probe Cs-corrected TEM (Titan cubed G2 60–300) operated at 300 kV. The Cs value was adjusted to be smaller than 200 nm. The C5 parameter was tuned by the manufacturer to be close to 400 µm. These microscope parameters were also used for the image simulations. Before STEM work, the TEM specimens were treated in a plasma cleaner for 10 min with a H_2_/O_2_ plasma recipe. All STEM images were slightly digitally filtered by a radial difference filter for noise reduction. Annular ranges of 79.5–200 mrad for the HAADF detector and 19–106 mrad for the low-angle ADF (LAADF) detector were applied. A probe forming aperture of 25 mrad was used in the HAADF and LAADF experiments. Image simulations were performed with the xHREM/xSTEM software package, based on the Fast Fourier Transform (FFT) multislice algorithm. The probe size was set to 0.07 nm which corresponds to the specified spatial resolution of the STEM instrument as identified from FFT image calculated from a high-resolution HAADF-STEM micrograph of GaN w-GaN $\left[2\bar{1}\bar{1}0\right]$. 

## 4. Conclusions

Growth characteristics, microstructure, and defects of thin GaN films, which were directly deposited on 6H-SiC(0001) substrates by ion-beam assisted molecular beam epitaxy (IBA-MBE) using hyperthermal nitrogen ions, was studied. Combined in situ RHEED and in vacuo STM investigations revealed that the film growth mode can be controlled by varying the ion to atom ratio I/A. Accomplished 2D growth resulted in films characterized by a smooth topography, a high crystalline quality, low biaxial stress, and low defect density. As a consequence, coalescence thicknesses below 10 nm (~30 GaN monolayers) could be achieved. 

The misfit accommodation mechanism and structural defects in epitaxial GaN thin films deposited by ion-beam assisted molecular-beam epitaxy on super-polished 6H-SiC (0001) substrates are also reported. The misfit strain is relieved by the generation of edge dislocations in the first and second monolayers of GaN thin films and of misfit interfacial dislocations between the GaN thin films and 6H-SiC substrates, suggesting two possible mechanisms of misfit strain accommodation. The dislocations are formed during initial stage of thin film growth by the formation of coherent GaN/SiC interface due to pseuodomorphic growth of GaN as a result of the high mobility of Ga and N species on the SiC surface. Threading dislocations having various nucleation sources are, in addition, found in the GaN thin films. Typical structural defects in the GaN thin films were identified as basal plane stacking faults, low angle grain boundaries forming between w-GaN and z-GaN and twin boundaries. 

Thus, hyperthermal ion assistance during epitaxial growth can lead to thin GaN films of high quality.
